# Captain Edward Mills Grace

**Published:** 1944

**Authors:** 


### Obituary

WE regret to have to record the following casualties on Service :—

### EDWARD MILLS GRACE, M.B., Ch.B. (Bristol), Captain R.A.M.C.

Captain Edward Mills Grace, R.A.M.C., elder son of Dr. E. M. Grace, of Thornbury, who died in March of typhoid fever in Italy. He graduated M.B. in 1939 and had held resident appointments at the B.R.I. One of his first war experiences was that of bringing back wounded from Dunkirk. Like his forbears, Grace was an outstanding cricketer and a fine all-round athlete.

